# How using light touch immersion research revealed important insights into the lack of progress in malaria elimination in Eastern Indonesia

**DOI:** 10.1186/s12936-024-04865-7

**Published:** 2024-02-27

**Authors:** Dee Jupp, Sherria Ayuandini, Frisca Tobing, Denny Halim, Enny Kenangalem, Maria E. Sumiwi, Hellen D. Prameswari, Minerva Theodora, Hermawan Susanto, Riskha T. P. Dewi, Dedy Supriyanto, Bayu Kurnia, Mrunal Shetye, Ermi Ndoen, Yuka Onishi

**Affiliations:** 1 EMPATIKA, Jakarta, Indonesia; 2 Ministry of Health, Jakarta, Indonesia; 3 UNICEF Indonesia, Jakarta, Indonesia

**Keywords:** Malaria, Risk perception, Social determinants, Behavior change, Immersion research, Papua, Sumba, Nusa Tenggara Timur

## Abstract

**Background:**

By 2022, the Government of Indonesia had successfully eliminated malaria in 389 out of 514 districts but continues to face a challenge in Eastern Indonesia where 95% of the total 2021 malaria cases were reported from Papua, West Papua and Nusa Tenggara Timur provinces. There is an increased recognition that malaria elimination will require a better understanding of the human behavioural factors hindering malaria prevention and treatment, informed by local context and local practice.

**Methods:**

This research used a light-touch immersion research approach. Field researchers lived in communities over several days to gather data through informal conversations, group-based discussions using visual tools, participant observation and direct experience. The study was conducted in four high malaria endemic areas in Papua, West Papua, and Sumba Islands in Nusa Tenggara Timur.

**Results:**

The research highlights how people’s perception of malaria has changed since the introduction of effective treatment which, in turn, has contributed to a casual attitude towards early testing and adherence to malaria treatment. It also confirms that people rarely accept there is a link between mosquitoes and malaria based on their experience but nevertheless take precautions against the annoyance of mosquitoes. There is widespread recognition that babies and small children, elderly and incomers are more likely to be seriously affected by malaria and separately, more troubled by mosquitoes than indigenous adult populations. This is primarily explained by acclimatization and strong immune systems among the latter.

**Conclusions:**

Using immersion research enabled behaviour research within a naturalistic setting, which in turn enabled experiential-led analysis of findings and revealed previously unrecognized insights into attitudes towards malaria in Eastern Indonesia. The research provides explanations of people’s lack of motivation to consistently use bed nets, seek early diagnosis or complete courses of treatment. The felt concern for the wellbeing of vulnerable populations highlighted during light touch immersion provides an entry point for future social behaviour change communication interventions. Rather than trying to explain transmission to people who deny this connection, the research concludes that it may be better to focus separately on the two problems of malaria and mosquitoes (especially for vulnerable groups) thereby resonating with local people’s own experience and felt concerns.

## Background

The Government of Indonesia (GoI) first introduced the Malaria Eradication Command (KOPEM) in 1959. The current malaria elimination programme aims to achieve the goal of malaria elimination by 2030. The programme comprises a variety of supply-driven interventions, including prevention through the distribution of long-lasting insecticidal net (LLINs) and indoor residual spraying (IRS), access to accurate diagnosis and standardized treatment, and establishment of surveillance systems.

These interventions have contributed to Indonesia’s substantial progress to achieve the malaria elimination goal, with a decrease in national malaria incidence based on Annual Parasite Incidence (API) from 1.75 (2011) to 0.84 (2018) per 1,000 population [1] and halving of confirmed malaria cases between 2010 and 2020 [2]. Moreover, 76% of Indonesia’s population now lives in 389 malaria free districts, largely in Java and Bali. In 2021, the World Health Organization (WHO) estimated there were more than 2.1 million malaria cases and an estimated 700–2,160 malaria-related deaths in Indonesia. Given that only 304,607 malaria cases and 48 malaria-related deaths were confirmed and reported by public hospitals in that year, there is reason to believe many of Indonesia’s malaria cases are not detected [1].

Despite positive progress toward malaria elimination, the number of cases in Indonesia has seen an increasing trend since 2018 [3] and three eastern provinces, Papua, West Papua, and Nusa Tenggara Timur, continue to be affected by malaria and accounted for 95% of reported cases in 2021. This high prevalence is attributed to a number of factors, including the unique bionomics of mosquitoes in these areas (habitats and biting behaviour), lack of testing, low capacity of health services (services available, health providers skills, shortages of malaria drugs and rapid diagnostic tests), poor housing and livelihood related factors such as malaria transmission among migrant workers in forestry, mining and plantations [4].

The persistently high incidence of malaria in these areas highlights the need to better understand the drivers and develop innovative solutions to address them. While many previous malaria elimination interventions have relied on supply-side interventions and focused on environmental factors, there is an increasing understanding that these efforts should also account for people’s behaviours e.g., treatment-seeking behaviour and preventive practices [5, 6]. Previous studies also assert that malaria interventions will only be effective if people engage with them and use them appropriately, e.g., wide distribution of bed nets is not sufficient if people do not use them when sleeping [7]. This is supported by wider approaches, such as Social and Behaviour Change Communication and Human Centred Design that are often used in health programming, which highlight the importance of grounding programmes in an understanding of people’s perspectives and local context in order to achieve the intended results [8–10].

In Indonesia, there are limited qualitative studies on determinants of behaviour that shape how people prevent and treat malaria and how people engage with supply-side services and act on communication messages. Analysis typically focuses on factors associated with either knowledge, attitude, or practices related to malaria prevention and treatment through surveys. Studies around people’s knowledge, for instance, typically explore gaps related to causes, symptoms and transmission of malaria, and factors influencing this such as education, occupation, socioeconomic background [11–14]. Related to attitude, some statistical analyses have linked delays in treating malaria to economic background and treatment preference [15] and people’s origin [16]. While these studies have outlined people's knowledge, attitude, and practice related to malaria interventions, few closely examine how these factors come together to shape people’s behaviour in preventing and treating malaria. Surveys have also been criticized for their unreliability in assessing people’s values, attitudes and behaviours in particular for the absence of context, reliance on memory and adoption of psychological defense mechanisms inherent in self-reporting [17–19].

This paper aims to fill this gap by offering new insights to understand the determinants shaping people’s behaviour towards malaria by using a light-touch immersion approach involving direct experience of the context and engaging a wide range of people in situ to understand their perspectives. In particular, it attempts to explain why local people do not heed advice or follow treatment regimes in regard to malaria. In addition to contributing to the body of knowledge related to malaria in Indonesia through insights from these locations, this paper intends to contribute to wider practice by highlighting implications of these insights for malaria elimination programming and advocates wider application of immersion-like research to uncover human behavioural barriers to change.

## Methods

### Study locations

The study was conducted in January to February 2021 in eight villages/communities, two in each of four districts in Eastern Indonesia; Jayapura and Timika in Papua province, Manokwari in West Papua province and Southwest Sumba in Nusa Tenggara Timur province. All districts were purposely selected on the basis of being high endemic areas. The Indonesian Ministry of Health defines high endemic as an area with Annual Parasite Incidence (API) > 5. The API ranged from 13 (Sumba) to 544 (Timika) according to 2021 data [20] and, therefore, a high priority for elimination efforts undertaken by the Government of Indonesia.

**Fig.1:**
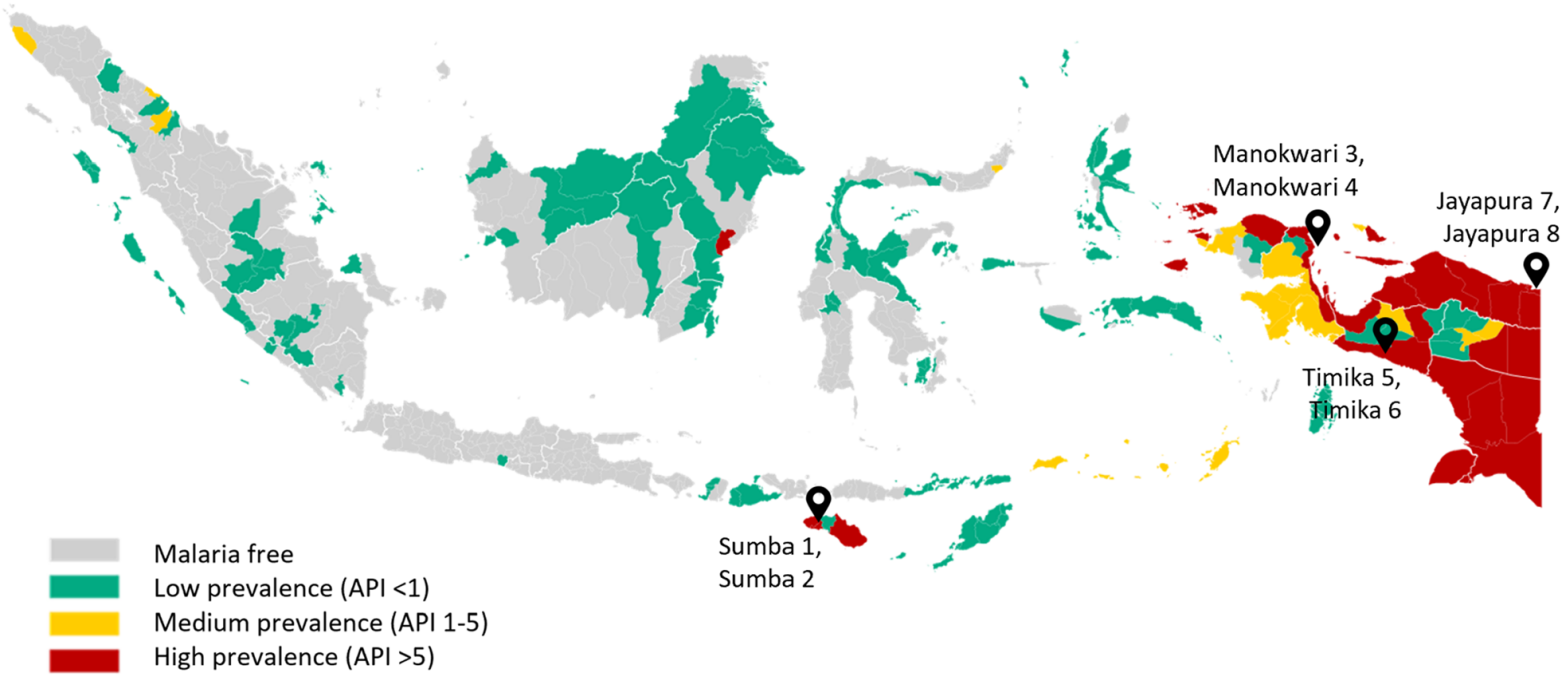
Study locations and prevalence of malaria (2022).

One urban location was selected in each district (Sumba 1, Manokwari 3, and Timika 5). One rural location was also selected in each district (Sumba 2, Manokwari 4, and Timika 6) while in Jayapura both locations selected were rural but represent very different topographies and livelihoods (lake fishing/agriculture in Jayapura 7 and agriculture/trading in Jayapura 8). To maintain the anonymity of these locations, villages are referred to by the district name and numbers used on the map (Fig. 1)﻿.

The villages range in size from around 500 inhabitants (Jayapura 7) to 6,000 inhabitants (Manokwari 3). Most people across all locations live in brick houses although Sumba was an exception where most live in traditional bamboo stilted houses with a few brick houses in the urban area (Sumba 1). In Manokwari, Timika and Jayapura districts the population is mostly Papuans but includes migrants from other parts of Indonesia especially in the urban locations. In Sumba the population comprises Sumba people (Sumba 1) and Kodi people (Sumba 2).

### Study participants

The study engaged entire households and families rather than individuals in order to understand the intersecting determinants affecting behaviour. Selection of the twenty four households that were the core focus research (three households per location) was purposeful and specifically included vulnerable individuals who have little or reduced immunity to malaria (pregnant women, infants under five years old, people with compromised immune systems), and those at risk from their livelihood choices (such as people migrating for work and those working in forestry or fishing). In addition to the core focus on households, field researchers engaged neighbours, service providers and other key influencers in the community.

The study included a total of 889 people across all eight villages. This equates to about 100 study participants per village and 30–35 participants engaged by each field researcher over the five days immersion. Most engagement involved conversations and observations but some involved observation only (see Table 1). Infants under five years old were only observed, for example whether they are sleeping/playing under bed nets, exposure to mosquitoes at different times of the day, how care-givers interacted with them to reduce risk and respond to illness. Interactions with other children included play and chat in order to gather insights into their perceptions on mosquitoes, risk and illness symptoms. The numbers by gender represent the actual numbers within focal households and neighbours as well as gender of health and service providers in particular areas.

**Table 1:** Summary of study participants (number and nature of participation)

	Nature of participation in study		Southwest Sumba		Manokwari		Timika		Jayapura	
	Observation	Conversation	M	F	M	F	M	F	M	F
Infant and children under 5	✓		5	7	5	9	4	3	5	5
Children (5–9 yo)	✓	✓	11	6	5	4	40	8	18	20
Adolescents (10–19 yo)	✓	✓	25	25	10	22	17	13	18	23
Adults and elderly (20 yo and above)	✓	✓	42	46	39	67	41	49	34	42
Pregnant women or recently pregnant women	✓	✓	–	5	–	6	–	6	–	2
Health providers e.g., traditional healer, doctor, midwives, nurse, and community volunteers		✓	11	1	6	9	6	22	0	6
Service providers e.g., teachers, NGO, village officials, faith leaders, kiosk owners, medicine sellers	✓ Traders only (kiosk owners, medicine sellers)	✓	7	11	27	20	29	19	12	16
Total participants			101	101	92	137	137	120	87	114

### Rationale for choice of method

The study of the determinants of behaviour change is challenging not least of all because behaviour is complex. As pointed out by Kelly and Barker [21], behavioural research requires a ‘deep understanding of what motivates people and the social and economic pressures that act upon them’. Although many have used surveys to explain people’s behaviours and attitudes, they have been criticized for lack of context and unreliability in self-reporting [17–19]. Qualitative methods are considered better at providing in-depth understanding of people’s experience and insights into their values, motivations, attitudes and behaviour [22, 23] These include interviews, focus groups and case studies which generally rely on the use of ‘invited spaces’ [24]. When people are invited to participate in what is effectively the expert space, a number of challenges arise which limit the authenticity of the voices and their experience. Furthermore, it is difficult to understand multiple perspectives when some people are never invited to, self-excluded from, or cannot access this space [25, 26]. Jupp further notes that such ‘invited spaces’ may be ‘constrained by power differences, vested interests, social desirability and sponsor biases’ and are often detached from context. As the study was specifically designed to understand why communities and individuals did not follow advice related to malaria prevention in endemic areas, the research method was selected to address the limitations of other methods of inquiry noted above and enable a deep contextual engagement and adopt a grounded theory approach. The approach lets emerging data speak for themselves rather than using external constructs to frame the research. This study was, therefore, designed to provide an alternative means to engage people in their own space (this comprises i. physical space where people feel comfortable and at ease, ii. conversational space where people can talk freely and lead the direction of conversations and iii. experiential space where people can augment what they say by showing and sharing their experiences [25]) in order to enable a broad range of people to participate in the study and enable an ethnographic approach which includes conversation, participant observation within a naturalist context. In particular, the choice of method was designed to generate emic [27] views of what is relevant, valued and significant.

The methodology used an internationally recognized light-touch immersion approach [25] whereby field researchers live with families and experience their everyday lives, interacting with all members of the family, their neighbours and others they naturally engage with. The approach builds on and extends the tradition of listening studies [28, 29] and beneficiary assessments [30] by combining elements of these approaches with field researchers actually experiencing context and using this shared experience to trigger conversation. As this study took place during the COVID-19 pandemic it was not possible to stay overnight in the families’ homes but researchers spent long days (usually 12 hours per day over 5 days) engaging with study participants.

The main means of engagement was through informal conversations which drew on topic guidelines prepared before the field work. These topic guidelines identified gaps in knowledge from the desk review [5] as well as gaps identified during a participatory workshop with the field researchers. The topic guidelines provided a point of reference for trained field researchers while also providing the needed flexibility when engaging with study participants to enable natural conversations rather than a prescribed set of interview questions.

The study team comprised 12 field researchers consisting 4 male and 8 female trained researchers with past experience of immersion research and participant observation in public health topics.

### Data collection

Each field researcher had previous direct experience of using immersion research in Indonesia, with most of them having used this approach in other countries too. In addition to training on how to collect data through discussions and observations through relaxed forms of interaction with study participants, good practice requires exercise of reflexivity, understanding and mitigating bias, maintaining informality and ethical considerations in conducting qualitative field research. Three field researchers worked independently of each other in each of the study villages and interacted with different people in different spaces.

Data were mainly collected through conversations, participant observations and some group-based interactions, such as mapping of mosquito breeding sites, recreational sites; seasonality diagrams to identify seasonal fluctuations in climate, incidence of illness, livelihoods; daily activity charts to gather insights into behaviour around perceived risk of malaria infection; ranking of common ailments by frequency and perceived severity; and, body mapping to understand how people identified symptoms of malaria and other diseases. This information was augmented by photographs and people’s interpretation of photographs. As researchers experienced the community context first-hand, this provided insights into risk factors and social norms and the accessibility of malaria preventative and curative services.

Field researchers shared their findings during intensive two-day de-briefing workshops immediately after completion of the 5-day immersion using the topic guidelines as a framework. The quality of data was assured through careful triangulation of data collected by different field researchers, of the different methods used and the different study participants. The workshops were led by research supervisors who questioned potential bias, encouraged reflexivity and ensured that multiple perspectives were all recorded. Recalled conversations, experiences and observations were recorded in detail in written and coded de-briefing notes. Detailed notes documenting this debriefing, along with photographs, annotated visual exercises, socio-economic templates for communities, written short case stories and field diaries from the immersion formed the ‘data set’ or basis of information from which study findings were drawn.

### Analysis

Analysis of the findings followed a ‘grounded theory’ approach. The established approach of Framework Analysis was used to examine the large quantity of observational and conversational data from the fieldwork (data-set) and included the following steps: i. Familiarization which involved detailed scrutiny of the de-briefing notes, re-reading case studies, field researcher team descriptions of individuals, households and communities; ii. Identification of thematic framework which involved identifying key issues, themes and categories raised through interaction with study participants emerging from the familiarization phase; iii. Interpretation which involved drawing inferences from the charted summaries. This process was undertaken by the research lead, co-lead, and Technical Adviser, both independently and jointly to identify and test emerging themes and findings. Working independently added credibility and rigour (i.e., different field researchers come to the same conclusions from the same written material/data set). Multilayered quality assurance was carried out through internal peer reviews, with special concern given to ensuring that the research retained the perspectives of the study participants, and other people with whom the field researchers engaged. Efforts were made to convey peoples’ voices rather than interpret people’s voices [25].

The study protocol was approved by the ethics review committee of the Institute of Research and Community Service of University Atma Jaya, Indonesia. All field researchers were briefed on ethical considerations (including Child Protection) before data collection commenced and signed a Code of Conduct on Confidentiality. Informed verbal consent was obtained and archived from all study participants or their guardians for using their experiences and insights for study reports and publications.

### Study limitations

Light-touch immersion research as discussed above, should provide some comparative advantage over other qualitative methods in that it takes place in peoples ‘own space’ and informally enables participation of people who otherwise may not participate. The inclusion of first-hand contextual experience is crucial. However, it does not provide the same depth of experience as longer ethnographic studies and is constrained by the timing of the study. This study was not undertaken at a time of high prevalence of malaria and so the observations and conversations may not reflect seasonal differences in behaviour. Furthermore, because this study was conducted during the COVID-19 pandemic, overnight stays in the villages were not permitted, limiting observation of overnight behaviour.

The approach relies considerably on the experience of the field researchers to build rapport, enable open and frank conversation, follow up on conversations and triangulate information during their field period. Most conversations are opportunistic and as not pre-arranged some service providers, for example, may not have been available at the time of the study.

## Results

Where there was a high level of convergence in views across different study villages and across genders and ages the subject is given the generalized term ‘people’ and refers specifically to community people. Where views differ, specific qualifiers are used to describe the study participants. Unless specified, the study findings below apply to all of the study locations.

### Perception of malaria

#### Perception has changed over time

Using visualized timeline diagrams which prompted recall of the past through talking with family and neighbour groups comprising different generations, people shared how their perception of and attitudes towards malaria have changed over time. Older people drew a particular distinction between the 1980’s and 1990’s. Before the 1990s, malaria was perceived as *‘rampant’*, deadly and extremely serious. Many remembered experiencing severe symptoms themselves and deaths among family and friends. The only treatments were herbal and traditional medicines. At this time Government incentives were provided to encourage families to migrate to Eastern Indonesia for work (Transmigration Programme) and people shared that these new migrants were particularly vulnerable to malaria. In the 1990s and continuing into the 2000s, the Government of Indonesia embarked on a major programme of construction of local health facilities; malaria medication (quinine pills) [31] became widely available and some malaria testing commenced in some locations such as Timika 4 and 5. People noted that there were fewer malaria deaths in the last decades [20], both among the local populations and incoming migrant communities.

*Without necessarily having an explanation, people shared that the incidence of malaria had dropped* with most adults saying they were not aware of getting it any more while children and adolescents were expected to get malaria at least once per year, some as many as five times per year. The local health facilities records across all study locations confirmed the perception that the incidence was declining. People did not believe that malaria would ever be eliminated but indicated that they felt less fearful and more supported to treat malaria.

#### Contemporary perception of seriousness of illnesses

Through asking older people to share personal stories of their own experience with malaria in the past it became clear that malaria was considered extremely serious in the past. By comparison, through co-generation of lists of diseases and ailments which concerned people now malaria was rarely mentioned unless prompted. Across study locations, people of all ages shared their concerned about diseases with severe consequences and risk of death, those that needed surgery, and that were perceived as expensive to treat such as heart disease, hypertension, diabetes, hypercholesterolaemia—especially about the need to take medication over long periods, cancer, filariasis, and HIV/AIDs [also see 32]. In Sumba 1 people worried about mouth cancer and spoke about it constantly. People were particularly concerned about long term debilitating effects of diseases which in turn affected their ability to earn income. TB carries social stigma and people in Sumba, where TB is common, shared their shame and concern regarding spreading this disease to others. Illnesses which were frequent such as respiratory infections were said to be more concerning than malaria, especially when children suffered regular infections.

Conversations explored these ailments listed as serious and the reasons for their emergence as concerns led to the recognition that there continues to be a strong belief in ‘suanggi’ or evil (revengeful) spirit which is used by people to explain why some contract a disease and others do not. As found in other studies [33], children’s ailments are often viewed as normal milestones in growing up, for example teething or learning to crawl is associated with a high chance that the child will experience a fever at that time. Parents are worried about fevers in very small children as they have undeveloped immune systems and they are aware that a fever may become very serious and is linked to seizures but nevertheless these are common and therefore somewhat normalized. The assumption that children must experience fevers regularly throughout their early years and the connection with childhood milestones, changes in season and ‘suanggi’ help explain why malaria is often regarded as just another fever and treatment is only sought if the fever does not subside over several days.

Typical of others interacted with, a mother whose 6 year old child had malaria contrasted it with dengue fever, said “*[With malaria we are] used to getting it often. … Dengue is dangerous because we don’t know about [it]. [We’re] more afraid of this,*” (woman, 40s, Jayapura 8) and there is no medicine.

Just a few people from Sumba 2, Jayapura 8, and Manokwari 3 described malaria as serious because of its symptoms and potential to cause brain damage and death but they knew through direct or indirect experience that effective malaria medication is now easily obtainable, which cures the symptoms quickly with infrequent recurrences.

#### Perception of malaria tolerance

Field researchers often had to specifically prompt discussion on malaria as it was not included in people’s lists of serious illnesses. As noted above, everyone had had personal experience of contracting malaria at some time in their lives. Some people used malaria susceptibility as an indicator of acclimatization which explained their experience that migrants, incomers and young children were more at risk. Incomers themselves concurred with comments such as; “*If you haven’t gotten malaria yet, you are not yet a Timika native,*” (man, 40s, Timika 5); “*It’s an introductory illness;*” (woman, 50s, Manokwari 4). Papuan study participants continually pointed to their resilience (‘*we are used to it’*) even noting the difference between Papuan and incomer teenagers as exemplified in the following ‘*a kid with level 4 malaria will play football while waiting for the (*test*) result but an incomer with level 1 wont be able to move.’*

To conclude, the confluence of perceptions that malaria is less risky, less prevalent and less severe than the past together with the assumption of acquired tolerance and the existence of effective cure has meant that malaria is now regarded as “*biasa aja*” (nothing out of ordinary). This acceptance was expressed in a number of different ways, “*We and malaria are already friends, becoming one,*” (people in Jayapura 8), “[Malaria is] *like my favorite warung nasi (eating place),*” (man, 70, Timika 5).

#### Local health service providers explanation of changed perception

Local health service providers explained the decline in incidence and reduction in serious consequences of malaria as due to the recent malaria elimination drive. They noted in particular increased bed net distribution (annual replacement), intermittent internal residual spraying and the introduction of dihydroartemisinin-piperaquine (DHP) and primaquine pills in 2007–2008. They also pointed out that since the 2010s many private and foundation-based malaria programmes have been initiated to complement the Government malaria elimination efforts. More recently, the Government has deployed local-level dedicated malaria cadres and increased capacity for malaria testing. Those health providers who participated in the study did not know that their communities did not consider malaria as a serious disease. Rather they blamed the lack of people’s concern with prevention, delayed treatment-seeking behaviour and non-completion of courses of treatment on people’s ignorance, laziness and stubbornness.

### Knowledge of malaria and its causes

*‘Everyone has had malaria—it does not discriminate by age, money or house’* (men, Sumba 2).

#### Community perceptions

People considered themselves knowledgeable about malaria. All those engaged with during the course of the light-touch immersion had had direct personal experience of malaria and adults were familiar enough with the symptoms and course of the illness to distinguish it from other illnesses. Most knew there were two types of malaria endemic in their area and could name them: malaria tropica and malaria tertiana. Personal experience enabled people to describe symptoms well and make distinctions between malaria and other diseases. Through drawing body maps, people explained in detail where pain was felt and the typical course of the illness. They noted high fever with many adding that body temperatures typically increased quickly, accompanied by severe headaches with shivering, muscle/body pain, feeling weak and vomiting.

Recognizing that malaria with this severity of symptoms is experienced rarely among adults nowadays and is associated with a lack of acclimatization, people ascribed a variety of reasons why some who would have been expected to be acclimatized nevertheless were badly affected by malaria. A weak or weakened immune system (primarily from fatigue, stress or not eating well) was the most common reason. Getting soaked in the rain or bathing in the river (particularly in the case of children), spending a lot of time outside (especially bare-chested), drinking coconut water, untreated water, and iced drinks were all linked to increased susceptibility to malaria. Others noted that blocked drains and a dirty environment also affected one’s ability to stave off infection, “*Trash impacts your immunity,*” (man, 30s, Timika 5). People also pointed to certain occupations, such as working in the forest and fishing, as demanding and tiring and, therefore, also likely to increase one’s susceptibility to illness including malaria.

With the belief that those who are weak and having low ‘immunity’ are the only ones for whom malaria may be a risk, people linked having a strong body with malaria prevention. For example, people in Jayapura 8 said they eat well and take enough rest to make their body stronger against malaria. Intensive activity is believed to actively “sweat out malaria”, for example “*When I work hard the malaria will come out of my pores like sweat,*” (man, 30s, Timika 6). During the immersion, field researchers examined the records at health centers and although women and children were more likely to have sought treatment, this was explained because indigenous men were more likely to think they were strong enough to overcome it without medication. People also recommended eating hot or spicy food to flush malaria out.

The idea that susceptibility has to be explained for a population who otherwise regard themselves as acclimatized also extends to perceptions of causal factors related to geography, weather and seasonality. Cold, rainy weather, rapid changes of weather were blamed for rises in malaria infections (as visualized using seasonality diagrams) and people were consistent in noting that malaria occurred most commonly during the rainy season. People also noted higher prevalence during fruit seasons such as mango season, and suggested that eating too much fruit may make people vulnerable to malaria. “*Probably from eating the fruit … too much mango,*” (woman, 30s, Manokwari 4) or that rising sap somehow affects vulnerability. Living in a mountainous area, on the coast, or nearby the forest, as well as in areas with plenty of trees and bushes were also noted as increasing risk for malaria.

Explaining why babies might be susceptible to malaria, people suggested the disease was passed on from mothers to children through pregnancy or breastfeeding. This chimes with the idea that adults are acclimatized but harbour malaria within their bodies. A few suggested that malaria was in the spleen, inside the heart, or in the blood and indicated this on their body maps.

In summary, malaria is perceived as something which weak or non-acclimatized people get. It is attributable to a variety of factors which are often framed as causes including weak immunity, climate, diet, weakness and most importantly lack of acclimatization. The perceived random and irregular incidence of malaria suggests to people that malaria is non-transmissible as it does not present as a typical transmissible disease, such as tuberculosis (TB).

### Delays in health-seeking behaviour for malaria

Using timelines and decision charting diagrams, people described their typical response to potential malaria. Across all study locations, the timelines revealed that people wait and observe any fever for a few days before visiting health providers. As noted above, people can often recognize the signs that their fever may be malaria and generally treated this themselves without seeking malaria diagnosis. They purchase non-prescription medicines from small vendors or local pharmacies and take these medicines until the symptoms subside.

Where people are less sure if they have malaria or not, they wait from between two to three days to a week before seeking medical care. During this time, they treat their fever and headaches with paracetamol and/or local herbal remedies. These local remedies include consuming bitter plants to cure fever or to support their immune system. These remedies were described in detail and field researchers were shown where plants were grown or gathered. Certain parts of these plants are eaten after boiling them, such as the leaves, including *sambiloto*, papaya, *kumis kucing*, bitter melon, *kelor* (moringa), *meniran*, itchy leaves, yellow, *awok*, *afrika*, *gamal*, *jambu biji* (guava), root, sap, flowers, bark, or fruits (solanacea berries, mahogany roots, ello tree bark, *langsat*). People also use *ungkup* or steam treatment, covering themselves with a blanket over boiling leaves to induce a sweat and ‘draw the fever’.

As noted above men prefer to try to fight the fever themselves, treating with local herbal remedies where needed and ‘sweating it out’. This approach is strongly linked to concepts of acclimatization and strength and, in turn, pride that they can master fevers. Only when the illness becomes very serious will men consider seeking medical advice. Some men and women explained the delay in seeking help from local health service providers was because they had been advised to wait because the malaria test might show false negative if done too early.

As noted above parents expect young growing children to have fevers often and mostly these are unrelated to malaria. The wait and see approach is the norm.

The study found a number of cases where families were convinced their sick relative had malaria but the tests were negative. They sometimes insisted on repeat tests and were vindicated by subsequent positive tests. The consequent distrust of the accuracy of the test as well as the long queues experienced by some trying to get tests, the delays in getting test results and the limited opening hours (especially of Government testing centres) led people to self-medicate with local remedies or malaria medication purchased over-the-counter.

### Perceptions of malaria treatment

As confirmed in timeline diagrams and through observation of blister packs containing unused tablets in people’s homes, medications were generally only taken until the symptoms subsided and people begin to feel better. This was irrespective of whether people choose to treat their suspected malaria themselves or get tested at a local health facility where they had been explicitly told to complete the course of medication. This behaviour was found more commonly among men as well as adolescent boys who usually finished their medication only when supervised by their mothers.

Those given medication from local health facilities not only discontinued taking this as they started to feel better but also shared that they experienced unpleasant side effects including buzzing ears, loss of hearing, severe headache, and drowsiness and were eager to discontinue as soon as possible. Some shared that the bitterness of the pills made them feel nauseous or made them actually vomit. Leftover pills were frequently kept to treat recurrence of symptoms or symptoms in other family members.

### Malaria transmission

#### Even though there is no perceived connection with malaria, mosquitoes are a problem

None of the explanations provided by people specifically links malaria to mosquitoes, although to an external observer many are of course linked with mosquito breeding seasons and behaviours. Only in Manokwari 4, Timika 6, and Sumba 2 was there any mention of mosquitoes having a part to play in transmission of malaria. Here a few people linked places with higher prevalence of mosquitoes to catching malaria but also knew that only some mosquitoes carry the disease. Even with this understanding only very few people had any idea that this involved blood transmission from person to person via mosquitoes.

When challenged to consider a possible link between mosquitoes and malaria during informal conversations, people often flatly denied this possibility. For example, Papuans shared that they knew people had malaria in Sulawesi but argued that mosquitoes could not fly that far. “*People don’t take the mosquitoes with them*,” (men and women, Jayapura 8). Typically, people argued that they frequently got bitten by mosquitoes but did not contract malaria whereas ‘*if you get soaked at night, you’ll get malaria in the morning,*” (man, 30s, Timika 5). Men in Sumba who work in the forest everyday shared they don’t bother to cover up and they get bitten by mosquitoes ‘*all over, but we’re not worried’*. Others shared that even using bed nets, ‘*we can still get malaria’* so they were unconvinced of any connection.

People noted that mosquitoes are a nuisance but they nevertheless put up with them, especially when they were busy working. “*You see the cashews ready for harvest, then forget about the mosquitoes, all you see is the money,*” (people in Sumba 2). People were particularly annoyed about mosquitoes disturbing their sleep. People shared through daily activities diagrams and seasonality diagrams that mosquitoes were more active during the afternoon, evenings, just before dusk and a few hours after dawn, as well as during rainy seasons and fruit seasons.

People across study locations were also very familiar with where mosquitoes were more prevalent such as puddles, fields, garden, bushes, forest, river, swamp, and beach. They drew maps and showed field researchers where there were ‘hot spots’ and many parents advised their children not to play in these areas. They compared these places to inside their houses, which they noted had ‘no mosquitoes’. It is important to note that people used the phrase ‘no mosquitoes’ to mean that there were not as many mosquitoes as researchers always observed mosquitoes inside houses. The key issue for people is not presence or absence but the nuisance created by hordes of mosquitoes. As a result, precautions were only observed in places where the density of mosquitoes was considered intolerable (forest, farm and bushland) while small numbers were slapped away. Only where people anticipate large quantities of mosquitoes do they use repellent, wear long sleeves or use smoke to repel them. They also took and used bed nets when having to stay in the forest for a certain period of time.

At home, there was less concern about mosquitoes. Field researchers observed bed nets in most of the houses visited and these were generally used frequently with people pointing out they were especially used during the fruit season and after rain. Some people did not use bed nets at all if there were few mosquitoes around, particularly those living in brick houses. “*I still have 20 bed nets. [I don’t use them] because [there are] no mosquitoes here,*” (woman, 60s, Timika 5). People shared that the main reasons for using bed nets were to keep mosquitoes from disturbing sleep, protection from wind and keeping warm.*’Nets are for a good night’s sleep’* (women, Sumba 2) and ‘*they are given away free so we have the opportunity for restful nights’* (people in Timika 6). Their use was not connected to malaria prevention except by a couple of elderly people who used bed nets as a habit formed when they were younger as a result of experiencing severe bouts of malaria.

This and other immersion studies noted that bed nets were more likely to be used for babies and toddlers [34–37], even during their day time naps. Electric fans where available were observed trained on sleeping infants to keep mosquitoes away. The concern was mainly to help babies sleep undisturbed by mosquitoes but also parents recognize that bites are more irritating for babies than adults which as well as disturbing sleep also became a source of skin infection.

Women and girls were more likely to sleep under nets partly because boys and men prefer to sleep outside and like to talk into the night. However, the nets used by women are often not tucked in because they restrict ease of getting up for the toilet at night.

### Local health service provider perceptions

Concerningly, many local health service providers did not know how malaria transmission occurs and their understanding of the ‘causes’ of malaria was often similar to that of community people. There was a greater appreciation of the link with mosquitoes and the need to prevent mosquito bites but susceptibility and seriousness of the disease was also linked to weak immune systems. “*Once bitten you will get it again if you eat irregularly, [if your] immune system is weak and don’t sleep under the bed net,*” (nurse in charge of the malaria programme, 40s, Manokwari 4). The community health volunteers in Sumba 1 advised community members not to drink untreated water as it made the body weak and enabled dormant malaria to become symptomatic. Other local service providers may know the transmission routes but found it easier to provide direct messages to the community. For example, one community health volunteer simply told people that, “*In Java, puddles cause dengue*” (woman, 50s, Timika 5). Another doctor also noted that it was fruitless trying to explain the mosquito connection with malaria and preferred to encourage people to maintain a strong immune system through eating well.

## Discussion

Malaria elimination has reached a plateau especially in Eastern Indonesia. This study was designed to gather insights into human behavioural factors which might contribute to this. It used light-touch immersion research techniques including informal conversation and engagement within shared contexts to help to understand people’s motivation and capability for behaviour change. Like others, this study advocates for behaviour research within naturalistic settings. While some researchers encourage greater use of informal conversations in qualitative research to ‘produce naturalistic data’ [38], this research augments this with participant observation, visualized exercises and interaction with all members of a household as well as their neighbours and service providers they interact with in the course of ordinary days. Using this approach enabled an experiential-led analysis of findings and a different perspective on what is hindering behaviour change.

The findings clearly indicate that people in the study areas know a lot about malaria but that they do not perceive it as a serious threat to themselves as they had in the past. As noted in other studies in Indonesia, malaria has been normalized as a fact of everyday life [16]. One could argue that efforts towards elimination have become the victim of their own success as increasing use of bed nets and IRS have reduced incidence and early testing and provision of effective medication has reduced mortality, leaving people with the perception of much reduced risk. With this risk de-escalation, people in high endemic areas such as in this study have acquired partial immunity from recovering from repeated bouts of malaria and describe themselves as strong, resistant and adapted to their environment. This further emphasizes their perception that only weak or those unfamiliar with the environment will succumb. Their own current experience of malaria is often mild (even asymptomatic) for which they see no reason to seek medical intervention. If people do not perceive malaria as a risk, experience mild symptoms or mistake these for seasonal ailments then they either do not seek treatment or delay treatment until symptoms become severe [39, 40].

Another key insight is that mosquitoes are primarily regarded as a nuisance and not a malaria vector. This is not a new finding but the study indicates that there is no action taken in regard to mosquitoes with an intention to reduce malaria infection. Like the perception of malaria itself, many regard the ability to put up with mosquitoes in terms of inurement. People focus on chores and livelihoods and regard mosquitoes as a nuisance only. They feel that a lifetime exposure to mosquitoes has reduced the irritant effect of bites. Observations during the study found that very few people took any precautions to protect themselves from mosquito bites by covering arms and legs, applying repellents or other measures to avoid bites and that sleeping under nets was primarily to avoid being kept awake by buzzing. Prevention of mosquito bites was not connected to protection against malaria not least of all because the frequency of the former does not correlate for people with any patterns of the latter.

The analysis suggests that people have little motivation to seek malaria treatment or prevent mosquitoes because neither problem is perceived as worrisome. Both are framed as functions of acclimatization to the environment (in other words, exposure has lessened the effect and consequently the concern) and choice to do anything about them is primarily an individual preference. The usual behaviour change communication response to such findings would be expected to put in place communication strategies to build better understanding of the transmission routes and to make people more aware of these links and risks. However, the researchers concluded that as neither of the perceived unconnected problems of malaria or mosquitoes were given priority, calls for behaviour change would continue to not be supported by lack of motivation.

However, the study concurred with others [33, 36, 37] that there was widespread concern about the health of young children. Parents do worry about any childhood fever particularly those associated with loss of appetite, crying and the fear of seizures. Local mother and baby clinics already highlight to mothers the link between frequent fever (and concomitant loss of appetite) with stunting. Although fevers are regarded as a normal part of ‘growing up’, parents and caregivers are especially concerned because young children have not developed their own ‘protection’ or ‘strength’ to combat disease. It was shared frequently that young children suffer bouts of malaria more often (have obvious and sometimes severe symptoms) than adults. Similarly, this study and others found parents and caregivers were more cautious about babies and toddlers being bitten by mosquitoes because they have a stronger reaction to bites and the potential allergy reaction from the bites [41–43]. The study revealed that bed nets are mostly used for small children because their reaction to bites is more severe than for adults. Similar views about susceptibility to malaria were expressed about incomers (not used to it) and elderly or others with compromised immune systems (not strong enough to combat it). These explanations are understood and accepted by most people and, therefore, provide an entry point for making a special case for care of these groups.

The Government of Indonesia has stepped up efforts to eliminate malaria through a number of supply-side interventions such as improved surveillance, vector control, mass testing and mass drug administration in endemic areas. The strategy also highlights the need for early diagnosis and provision of complete and timely treatment. The needed complementary social and behaviour change communication efforts to achieve this are less successful and this study partly explains this by highlighting the lack of interest and motivation of residents of endemic areas who play down the seriousness of the problem. Under these circumstances a behaviour change communication approach which focuses on the acknowledged parental concern for babies and small children has a higher likelihood of traction. Parents and caregivers may be motivated by the recommendation that all children under five with even the mildest of fevers are tested for malaria immediately to rule out this possibility or to provide early diagnosis and treatment. Similarly, adults who do test positive for malaria can be encouraged to complete courses of medication in order to limit potential infection to children under five. By association, people are also likely to recognize the vulnerability of unborn children and take similar precautions around pregnant women. Addressing the two problems which matter to people – the risks of both malaria and mosquitoes for children under five – separately avoids trying to explain the transmission cycle which people find baffling and which seems to contradict their perception and experience of cause and effect.

## Conclusions

The use of light-touch immersion research provided an opportunity to engage people in conversations concerning their perceptions and attitudes towards malaria, something which they referred to as ‘a friend’ in that they have come to terms with living with it. The opportunity for field researchers to directly experience a shared context with people living in the high endemic study locations enabled the gathering of new insights and provided a different perspective on the problems. Triangulation of data from different methods (conversation, visualized joint analysis with study participants, observation and experience) as well as triangulation of data from different contexts, across different ages and gender, at different times of the day and evening enabled confidence in the findings. Putting people at ease through the use of informal means of engagement and interacting within their own everyday home and work environments contributed to understanding real-life decision making and practice. This often highlighted the difference between what people said they do (especially when challenged or checked up on by health service providers) and what they really do.

The conventional ‘knowledge, attitudes and practice’ lens for behaviour change posits the need for information and knowledge before practice change happens. However, the study points out that people do not accept information when it does not resonate with their experience, such as the case with malaria transmission. High exposure to mosquito bites (especially among men working in forests, for example) does not correlate with the perception of minimal incidence of malaria. The concept of carrying malaria asymptomatically is difficult to grasp. While the Government of Indonesia is addressing the opportunity to change through provision of bed nets, locally accessible diagnostic and treatment services as well as improving vector control and surveillance, the motivation to change behaviour to use bed nets properly, seek early diagnosis and complete courses of treatment remains with people themselves. Where people dispute the evidence and diminish the seriousness of the issue, motivation becomes an intransigent challenge.

As a result of the insights gained from this study it is suggested that rather than focusing on and trying to explain the science of transmission and risk, there may be value in focusing on issues which do concern people. In this case it is the genuine worry about childhood fevers and the knowledge that these are sometimes attributable to malaria which is perceived as more serious for young children. There is also concern about mosquitoes biting and disturbing the sleep of small children with the recognition that this is worse for children than adults.

People do not need to practice perfect behaviours or have complete knowledge (for example an understanding of the link between mosquitoes and malaria) for them to make important behaviour changes which will make a difference. Protect vulnerable from mosquito bites; seek early diagnosis for every childhood fever and complete any malaria treatment given are simple messages which this research has shown would resonate with the felt concerns of people.

## Data Availability

The datasets generated and/or analysed during the current study are not publicly available but are available from the corresponding author on reasonable request.

## Abbreviations

IRS: Indoor residual spraying
LLINs: Long-lasting insecticidal net
TB: Tuberculosis
WHO: World Health Organization

## References

[1] WHO (2018). Indonesia—Country Profile 2018.

[2] Kementerian Kesehatan. Hari Malaria Sedunia 2021. 2021. http://p2p.kemkes.go.id/hari-malaria-sedunia-tahun-2021/. Accessed 5 Dec 2023.

[3] World Bank. Incidence of malaria (per 1000 population at risk)—Indonesia. https://data.worldbank.org/indicator/SH.MLR.INCD.P3?contextual=default&end=2020&locations=ID&start=2016&view=chart. Accessed 4 Feb 2023.

[4] Gani A, Budiharsana MP. The consolidated report on Indonesia health sector review 2018—National Health System Strengthening. 2019. https://www.unicef.org/indonesia/reports/consolidated-report-indonesia-health-sector-review-2018. Accessed 29 Dec 2020.

[5] Ayuandini S, Jupp D, Tobing F. Social determinants influencing access to malaria services: a formative study in NTT, Papua, and West Papua. Jakarta; 2021. https://www.empatika.org/projects/social-determinants-influencing-access-to-malaria-services. Accessed 29 Nov 2023.

[6] Bannister-Tyrrell M, Verdonck K, Hausmann-Muela S, Gryseels C, Muela Ribera J, Peeters GK (2017). Defining micro-epidemiology for malaria elimination: systematic review and meta-analysis. Malar J.

[7] Monroe A, Olapeju B, Moore S, Hunter G, Merritt AP, Okumub F (2021). Improving malaria control by understanding human behaviour. Bull World Health Organ.

[8] Koenker H, Keating J, Alilio M, Acosta A, Lynch M, Nafo-Traore F (2014). Strategic roles for behaviour change communication in a changing malaria landscape. Malar J.

[9] Toso M. Social and behavior change considerations for areas transitioning from high and moderate to low, very low and zero malaria transmission. Health Communication Capacity Collaborative (Baltimore). 2017. https://healthcommcapacity.org/social-behavior-change-considerations-areas-transitioning-high-moderate-low-low-zero-malaria-transmission/. Accessed 30 Dec 2020.

[10] Johnson T, Sandhu JS, Tyler N. The next step for human-centered design in global public health [Internet]. 2019. Stanford Social Innovation Review. https://ssir.org/articles/entry/the_next_step_for_human_centered_design_in_global_public_health#. Accessed 30 Dec 2020.

[11] Sanjana P, Barcus MJ, Bangs MJ, Ompusunggu S, Elyazar I, Marwoto H (2006). Survey of community knowledge, attitudes, and practices during a malaria epidemic in Central Java. Indonesia Am J Trop Med Hyg.

[12] Finda MF, Moshi IR, Monroe A, Limwagu AJ, Nyoni AP, Swai JK (2019). Linking human behaviours and malaria vector biting risk in south-eastern Tanzania. PLoS ONE.

[13] Dhewantara PW, Ipa M, Widawati M (2019). Individual and contextual factors predicting self-reported malaria among adults in eastern Indonesia: findings from Indonesian community-based survey. Malar J.

[14] Ipa M, Widawati M, Laksono AD, Kusrini I, Dhewantara PW (2020). Variation of preventive practices and its association with malaria infection in eastern Indonesia: findings from community-based survey. PLoS ONE.

[15] Karyana M, Devine A, Kenangalem E, Burdarm L, Poespoprodjo JR, Vemuri R (2016). Treatment-seeking behaviour and associated costs for malaria in Papua. Indonesia Malar J.

[16] Rustam M. An evaluation of LLIN distribution Program in three regencies in Indonesia. Jakarta.

[17] Schwarz N (1999). Self-reports: How the questions shape the answers. Am Psychologist.

[18] Schwarz N (2007). Cognitive aspects of survey methodology. Appl Cogn Psychol.

[19] Hansen PG, Larsen EG, Gundersen CD (2022). Reporting on one’s behavior: a survey experiment on the nonvalidity of self-reported COVID-19 hygiene-relevant routine behaviors. Behavioural Public Policy.

[20] Ministry of Health. Situation Report on Malaria Control Programme in Indonesia Year 2021. Jakarta; 2021.

[21] Kelly MP, Barker M (2016). Why is changing health-related behaviour so difficult?. Public Health.

[22] Tenny S, Brannan JM, Brannan GD. Qualitative study. In: Treasure Island. StatPearls Publishing. 2022. https://www.ncbi.nlm.nih.gov/books/NBK470395/29262162

[23] Agius SJ (2013). Qualitative research: its value and applicability. Psychiatrist.

[24] Gaventa J (2006). Finding the spaces for change: a power analysis. IDS Bull.

[25] Jupp D, Burns D, Howard J, Ospina SM (2021). Reality check approach: immersion research. The SAGE handbook of participatory research and inquiry.

[26] Jupp D (2021). Using immersion research and people-driven design to improve behavior change programs. Int J Market Res.

[27] Given LM (2008). The SAGE encyclopedia of qualitative research methods.

[28] Anderson MB, Brown D, Jean I. Time to listen: hearing people on the receiving end of international aid - CDA Collaborative. Cambridge, Massachusetts: CDA Collaborative Learning Projects; 2012. https://www.cdacollaborative.org/publication/time-to-listen-hearing-people-on-the-receiving-end-of-international-aid/. Accessed 29 Nov 2023.

[29] Salmen LF. Toward a listening bank: a review of best practices and the efficacy of beneficiary assessment. Social Development Papers. 1998. https://www.betterevaluation.org/tools-resources/toward-listening-bank-review-best-practices-efficacy-beneficiary-assessment. Accessed 29 Nov 2023.

[30] Shutt C, Ruedin L. How-to-note beneficiary assessment (BA). Berne; 2013. https://www.participatorymethods.org/resource/sdc-how-note-beneficiary-assessment-ba. Accessed 29 Nov 2023.

[31] Fitri LE, Pawestri AR, Winaris N, Endharti AT, Khotimah ARH, Abidah HY (2023). Antimalarial drug resistance: a brief history of its spread in Indonesia. Drug Des Devel Ther.

[32] Mboi N, Syailendrawati R, Ostroff SM, Elyazar IRF, Glenn SD, Rachmawati T (2022). The state of health in Indonesia’s provinces, 1990–2019: a systematic analysis for the Global Burden of Disease Study 2019. Lancet Glob Health.

[33] Jupp D, Sugandi Y. Universal child grant baseline qualitative review. Jakarta; 2018.

[34] Reality Check Approach Ghana Team. Millennium villages evaluation: Endline reality check approach. Brighton; 2017. https://itad.com/wp-content/uploads/2018/09/2018_MVEval_Annex-C_RCA-Endline-Report_submitted_04dec17-ID-101242.pdf. Accessed 6 Dec 2023.

[35] Barnett C, Masset E, Dogbe T, Jupp D, Korboe D, Acharya A, et al. Impact evaluation of the SADA Millennium Villages project in Northern Ghana: endline summary report. Brighton; 2018. https://www.itad.com/knowledge-product/endline-summary-report-impact-evaluation-of-the-sada-millennium-villages-project-in-northern-ghana/. Accessed 6 Dec 2023.

[36] Empatika. Exploring Maternal, Infant, and Young Child Nutrition & Early Childhood Development Practices in Indonesia. Jakarta; 2019.

[37] Empatika. Exploratory Research Phase 1.1 Immersion for Better Investment for Stunting Alleviation (BISA). Jakarta; 2020.

[38] Swain J, King B (2022). Using informal conversations in qualitative research. Int J Qualitat Methods.

[39] Roosihermiatie B, Nishiyama M, Nakae K (2000). The human behavioral and socioeconomic determinants of malaria in Bacan Island, North Maluku. Indonesia J Epidemiol.

[40] Reality Check Approach Plus. Children and their families perspectives and experiences on poverty and social protection. Jakarta; 2016.

[41] Orimadegun AE, Ilesanmi KS (2015). Mothers’ understanding of childhood malaria and practices in rural communities of Ise-Orun, Nigeria: implications for malaria control. J Family Med Prim Care.

[42] Kio JO, Agbede CO, Olyinka FE, Omeonu PE, Dire-Arimoyo Y (2016). Knowledge, attitudes and practices of mothers of under-five regarding prevention of malaria in children: evidence from Ogun State. Nigeria IOSR J Humanities Soc Sci.

[43] Vander Does A, Labib A, Yosipovitch G (2022). Update on mosquito bite reaction: Itch and hypersensitivity, pathophysiology, prevention, and treatment. Front Immunol.

